# Co-crystal structure, Hirshfeld surface analysis and DFT studies of 3,4-ethyl­ene­dioxy­thio­phene solvated bis­[1,3-bis­(penta­fluoro­phen­yl)propane-1,3-dionato]copper(II)

**DOI:** 10.1107/S2056989020006155

**Published:** 2020-05-15

**Authors:** Yusuke Habuka, Emily Ami Takeuchi, Akiko Hori

**Affiliations:** a Department of Applied Chemistry, Graduate School of Engineering & Science, Shibaura Institute of Technology, 307 Fukasaku, Minuma-ku, Saitama-shi, Saitama 337-8570, Japan; b Center for Natural and Human Sciences (CCNH), Federal University of ABC, Santo Andre, Sao Paulo 09210-580, Brazil

**Keywords:** crystal structure, co-crystal, Hirshfeld surface analysis, 3,4-ethyl­ene­dioxy­thio­phene, EDOT

## Abstract

The title complex, Cu(*L*)_2_ or [Cu(C_15_HF_10_O_2_)_2_], comprised of one copper ion and two fully fluorinated ligands (*L* ^−^), was crystallized with 3,4-ethyl­ene­dioxy­thio­phene (EDOT, C_6_H_6_O_2_S) as a guest mol­ecule to give in a di­chloro­methane solution a unique co-crystal, Cu(*L*)_2_·3C_6_H_6_O_2_S. In the crystal, the oxygen of one guest mol­ecule, EDOT-1, is coordinated to the metal to give an alternate linear arrangement, and the π-planes of the others, EDOT-2 and EDOT-3, inter­act weakly with the penta­fluoro­phenyl groups of the complex through arene–perfluoro­arene inter­actions. Head-to-tail columnar and head-to-head dimeric arrangements are observed for EDOT-2 and EDOT-3, respectively, in the crystal. The Hirshfeld surface analysis indicated that the most important contributions for the crystal packing are from the F⋯F (20.4%), F⋯H/H⋯F (24.5%) and F⋯C/C⋯F (9.6%) inter­actions. The density functional theory (DFT) optimized structure at the ωB97X-D 6–31G* level was compared with the experimentally determined mol­ecular structure in the solid state.

## Abstract 2

The title complex, Cu(*L*)_2_ or [Cu(C_15_HF_10_O_2_)_2_], comprising one copper ion and two fully fluorinated ligands (*L* ^−^), was crystallized with 3,4-ethyl­ene­dioxy­thio­phene (EDOT, C_6_H_6_O_2_S) as a guest mol­ecule to give in a di­chloro­methane solution a unique co-crystal, Cu(*L*)_2_·3C_6_H_6_O_2_S.

## Chemical context   

3,4-Ethyl­ene­dioxy­thio­phene, EDOT, is a familiar reagent for polythio­phene or oligo­thio­phene organic-active materials such as organic conductive macromolecules and optoelectronic materials. The corresponding poly-3,4-ethyl­ene­dioxy­thio­phene, PEDOT, is one of the typical organic conductive materials with a high conductivity, environmental stability, mechanical strength and visible light transmittance, thus showing wide ranges of applications (Skotheim *et al.*, 1998 ▸; Groenendaal *et al.*, 2000 ▸; Kirchmeyer & Reuter, 2005 ▸). The affinity as a guest mol­ecule and the corresponding inter­molecular inter­actions in co-crystals of EDOT are crucial issues for chemists in order to understand the mol­ecular recognition and supra­molecular association events (Storsberg *et al.*, 2000 ▸). The crystal packing and the relative inter­molecular inter­actions are estimated by the oxygen and sulfur atoms for coordination bonds and mol­ecular stacking of the π-inter­actions for the five-membered hetero-conjugated aromatic ring. On the other hand, mol­ecular crystals of fully fluorinated coordination complexes have been studied as hosts, showing flexible and responsive crystal-packing structures depending on the guest mol­ecules. Typically, the copper complex, Cu(*L*)_2_, produces unique co-crystals abundantly taken into benzene derivatives after crystallization and reversibly encapsulates their vapors (Hori *et al.*, 2014 ▸), while the corresponding single crystals of Cu(dbm)_2_ (dbm = di­benzoyl­methane) showed no inter­action with the guest mol­ecules. The driving forces of the mol­ecular recognition estimated a metal⋯π inter­action (Hunter, 1994 ▸; Ma & Dougherty, 1997 ▸) induced by improvement of the cationic properties of the central metal as a result of the fluorine-withdrawing nature and arene–perfluoro­arene inter­action (Williams, 1993 ▸, 2017 ▸; Hori, 2012 ▸) induced by the exact opposite quadrupole moment between the penta­fluoro­phenyl ring of the complex and the aromatic ring of the guest mol­ecule.
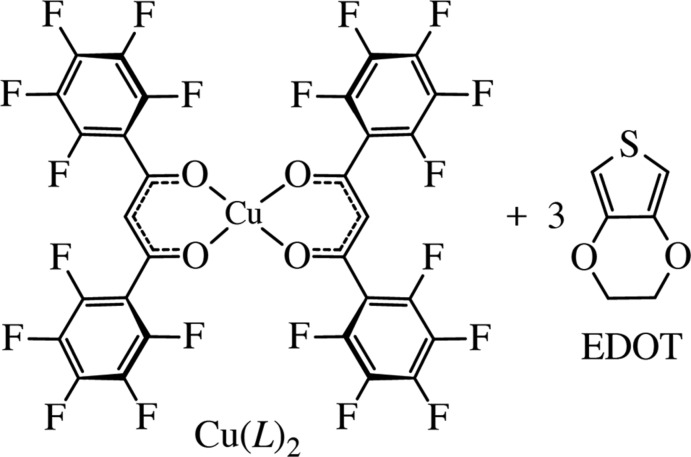


In this study, we examined the encapsulation of 3,4-ethyl­ene­dioxy­thio­phene for the title complex, Cu(*L*)_2_, indicating a new guest-encapsulated crystal, Cu(*L*)_2_·3EDOT (I), as shown in the Scheme. The crystal of (I) was prepared by previously reported protocols (Hori & Arii, 2007 ▸). Typically, Cu(*L*)_2_ and an excess amount of EDOT in CH_2_Cl_2_ (or AcOEt) were slowly evaporated to yield green block-shaped crystals. The driving forces and the detailed weak inter­molecular inter­actions were investigated by Hirshfeld surface analysis and DFT calculations. Using the same procedure, the corresponding compound Pd(*L*)_2_·*n*EDOT was not obtained, then Pd(*L*)_2_ was separately crystallized, showing different metal characteristics and affinity for EDOT. The electrostatic potential of the metal ions is also discussed.

## Structural commentary   

**Figure 1:**
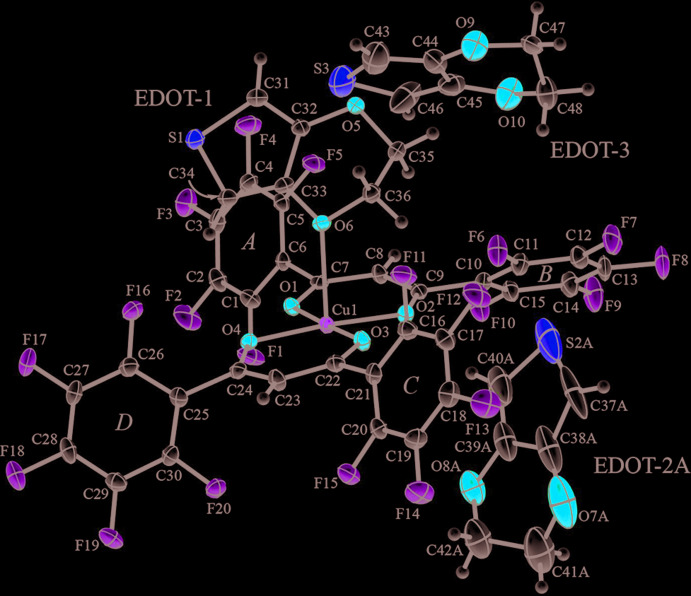
The mol­ecular structure of (I) at 100 K, showing the atom-labeling scheme. Displacement ellipsoids are drawn at the 50% probability level. The minor EDOT-2*B* component is omitted.

The asymmetric unit of (I) contains one entire complex mol­ecule and three EDOT mol­ecules. The complex is non-centrosymmetric and comprises one Cu^2+^ ion and two ligands (*L*) to give a mononuclear Cu^2+^ complex, as shown in Fig. 1 ▸. The geometry around the metal center is pseudo-square planar; the bond distances Cu1—O1, Cu—O2, Cu—O3 and Cu1—O4 are 1.940 (2), 1.941 (2), 1.922 (2) and 1.928 (2) Å, respectively. The penta­fluoro­phenyl groups [rings *A*–*D* (C1–C6, C10–C15, C16–C21 and C25–C30, respectively)] are highly twisted with respect to the coordination plane; the dihedral angle between ring *A* (or ring *B*) and Cu1/O1/C7–C9/O2 is 65.80 (13)° [or 36.24 (15)°] and the dihedral angle between ring *C* (or ring *D*) and Cu1/O3/C22–C24/O4 is 54.97 (14)° [or 51.22 (13)°], indicating that all these rings are crystallographically different. The flexible and twisted rings allow inter­molecular inter­actions with the EDOT mol­ecules to consolidate the crystal of (I). The oxygen atoms of EDOT-1 are coordinated with atom Cu1 of the complex mol­ecule; the lengths of the coordination bonds are 2.421 (2) and 2.711 (2) Å for Cu1—O6 and Cu1—O5^i^ [symmetry code: (i) *x* + 1, *y*, *z*], respectively (Figs. 1 ▸ and 2 ▸ *a*). The EDOT-2 mol­ecule shows disorder, the occupancy of the major component, EDOT-2*A*, being 0.691 (4); EDOT-2*A* shows close inter­actions with ring *C* of Cu(*L*)_2_ through an arene–perfluoro­arene inter­action. The EDOT-3 mol­ecule shows no remarkable inter­actions in the crystal packing as discussed below. Each EDOT mol­ecule shows a π-localized structure as shown in the Scheme; the lengths of the C=C double bonds are 1.355 (5) and 1.351 (4) Å for EDOT-1, 1.46 (1) and 1.32 (1) Å for EDOT-2*A*, and 1.361 (6) and 1.365 (6) Å for EDOT-3. EDOT-2*A* has a large variation in the distance because of the structural disorder, while the analysis was performed without restricting the binding distance of the carbon-to-carbon bonds. For comparison of the mol­ecular recognitions of Cu(*L*)_2_, negative quadrupole moments of the mol­ecules, *e.g.*, benzene and carbon dioxide, are reversibly recognized in the crystals, because of the positive quadrupole moments of the penta­flurophenyl groups (Hori *et al.* 2014 ▸, 2017 ▸). Thus, the crystal structure of (I) indicates the possibility that the butadiene moiety, C=C—C=C, in EDOT also has a negative surface and inter­acts in the crystal of Cu(*L*)_2_ through electrostatic inter­actions.

## Supra­molecular features   

**Figure 2:**
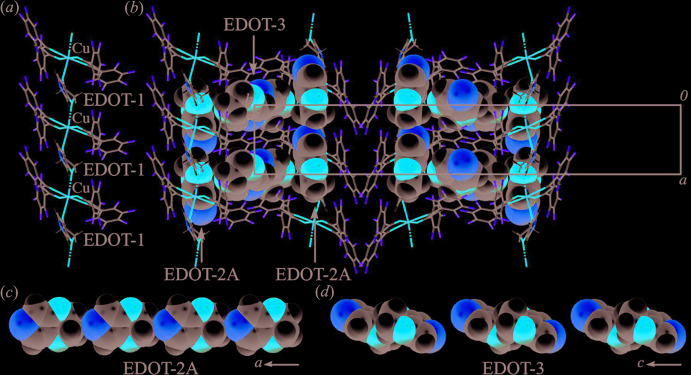
Views of part of the crystal structure of (I): (*a*) 1:1 alternating linear structure with EDOT-1 and Cu(*L*)_2_, (*b*) EDOT-2*A* and EDOT-3 in the void spaces of the linear chain with the (*c*) head-to-tail and (*d*) head-to-head arrangements in the crystal. Color scheme: C, gray; H, white; Cu, orange; F, light green; O, red; S, yellow.

The partial view of the packing structure in Fig. 2 ▸ *a* clearly shows a one-dimensional linear chain orientation between the complex mol­ecule and EDOT-1. EDOT-1 coordinates to the copper ion of the complex to form a 1:1 alternating linear structure along the *a-*axis direction. The EDOT-2*A* and EDOT-3 mol­ecules are inserted in the voids of the linear chain along the *a*- and *c*-axis directions, respectively. EDOT-2*A* forms a head-to-tail one-dimensional chain (Fig. 2 ▸ *c*) with weak hydrogen bonds (Table 1 ▸) between the sulfur atom and the aliphatic proton with *D*⋯*A* distances of 3.051 (11) and 3.220 (9) Å for C41*A*—H41*A*⋯S2*A* and C42*A*—H42*A*⋯S2*A*, respectively, and the mol­ecule is further sandwiched by the penta­fluoro­phenyl rings of the complex. EDOT-3 forms discrete dimers (Fig. 2 ▸ *d*) in a head-to-head configuration between the aliphatic moieties, and the dimers are also surrounded by the penta­fluoro­phenyl rings of the complex mol­ecule. Short inter­molecular inter­actions between the centroids (*Cg*) of the penta­fluoro­phenyl ring in Cu(*L*)_2_ and the five-membered ring of EDOT are observed. The penta­fluoro­phenyl ring *A* (C1–C6) is sited on the adjacent EDOT-2*A* ^ii^ (S2*A*/C37*A*–C40*A*) [symmetry code: (ii) *x*, *y*, *z* + 1]: the centroid–centroid distance *Cg*⋯*Cg* is 3.950 (4) Å and the shortest perpendicular distance of *Cg* (ring *A*) on the ring of EDOT-2*A* ^ii^ is 3.0832 (13) Å. Ring *B* (C10–C15) is sandwiched between two adjacent mol­ecules, EDOT-3^iii^ and EDOT-3^iv^ (S3/C43–C46) [symmetry code: (iii) −*x*, −*y* + 1, −*z* + 1; (iv) −*x* + 1, −*y* + 1, −*z* + 1]: the centroid–centroid distances are 3.906 (2) and 4.054 (2) Å, respectively, and the corresponding shortest perpendicular distances are 3.5236 (19) and 3.2687 (15) Å, respectively. Ring *C* (C16–C21) inter­acts with EDOT-2*A* (S2*A*/C37*A*–C40*A*) and EDOT-2*B* (minor disorder component; S2*B*/C37*B*–C40*B*); the centroid–centroid distances are 3.586 (3) and 3.684 (5) Å, respectively, and the corresponding shortest perpendicular distances are 3.5337 (14) and 3.299 (4) Å, respectively. Ring *D* (C25–C30) inter­acts with the adjacent EDOT-1^i^ (S1/C31–C34) with centroid–centroid and perpendicular distances of 3.7052 (19) and 3.3405 (13) Å, respectively. The results indicate that a remarkable arene–perfluoro­arene inter­action is observed for EDOT-2*A* with a length close to the sum of the van der Waals radii. A notable intra­molecular C—F⋯π inter­action is observed between F5 and EDOT-1 [3.287 (2) Å] and inter­molecular C—F⋯π inter­actions occur between the penta­fluoro­phenyl rings as an F⋯π(hole) inter­action; the distances are 2.997 (2) and 3.175 (3) Å for F9⋯ring *A* ^iv^ and F14⋯ring *D* ^v^, respectively [symmetry code: (v) *x*, −*y* + 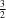, *z* − 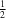]. These aromatic inter­actions are estimated to be induced by the positive electron distribution and quadrupole moment of the penta­fluoro­phenyl rings.

## Hirshfeld surface analysis   

**Figure 3:**
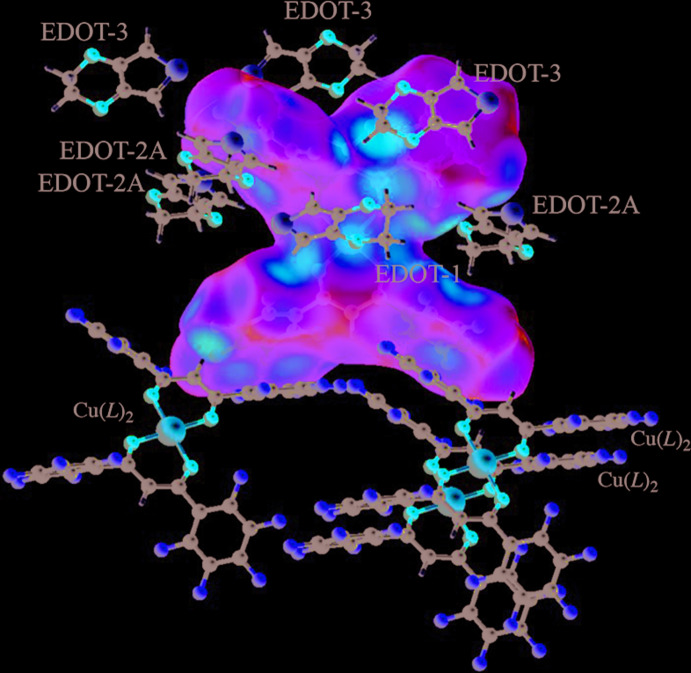
HS of the complex mapped with *d*_e_.

**Figure 4:**
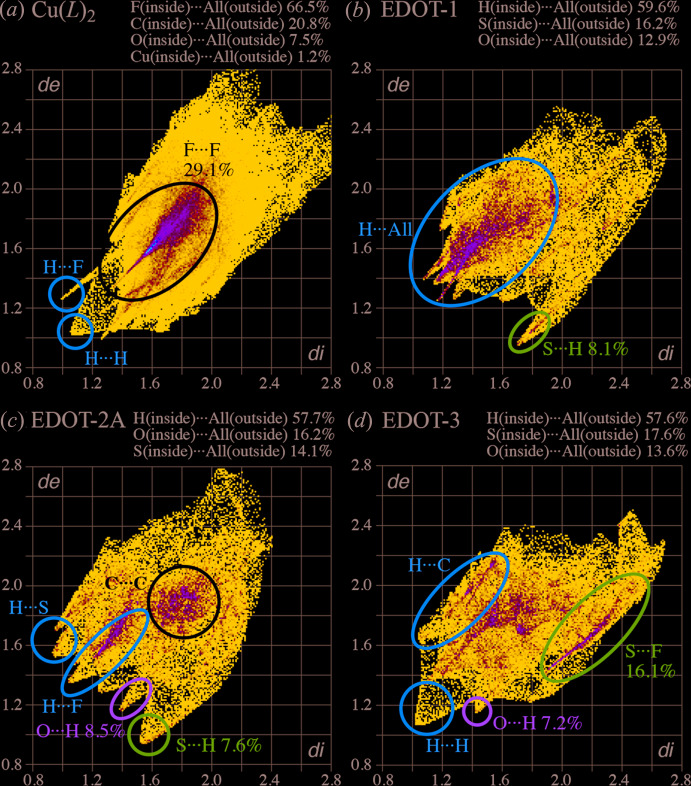
Fingerprint plots for the Cu(*L*)_2_ and EDOT mol­ecules in (I).

To understand all the inter­molecular inter­actions, a Hirshfeld surface (HS) analysis (Hirshfeld, 1977 ▸; Spackman & Jayatilaka, 2009 ▸) was carried out using *Crystal Explorer 17.5* (Turner *et al.*, 2017 ▸). The HS of the complex mol­ecule mapped with *d*_e_ (the distance between the surface and external atoms) and the corresponding fingerprint plots are shown in Figs. 3 ▸ and 4 ▸, respectively. The complex Cu(*L*)_2_ is surrounded by EDOT and Cu(*L*)_2_ mol­ecules and the inter­molecular inter­actions are indicated in red (Fig. 3 ▸). The main inter­actions for the whole structure are F⋯F and F⋯H/H⋯F, contributing 20.4% and 24.5%, respectively, to the overall crystal packing due to the high surface area of fluorine for the complex. The presence of π–π and C—H⋯π inter­actions is reflected in the contributions of the C⋯C (5.2%) and C⋯H/H⋯C (6.2%) contacts. The two-dimensional fingerprint plots (McKinnon *et al.*, 2007 ▸) of the independent Cu(*L*)_2_ and three EDOT mol­ecules are shown in Fig. 4 ▸ *a*–*d*, together with the contributions of each element. For Cu(*L*)_2_, the contribution of the Cu atom indicates interaction only with the oxygen of EDOT-1 (1.2%). For the three EDOT mol­ecules, the main inter­actions are H⋯F contributing 23.6%, 25.3%, and 26.8% for EDOT-1, 2*A* and 3, respectively. The contribution of the π–π inter­actions through C⋯C inter­actions shows the relationship EDOT-2*A* (8.2%) > EDOT-3 (6.0%) > EDOT-1 (4.5%), which indicates good agreement of the arene–perfluoro­arene inter­actions in the crystal packing. For the sulfur in EDOT, the S⋯H inter­action is observed for EDOT-1 (8.1%) > EDOT-2*A* (7.6%), but no inter­action for EDOT-3 (0.0%) and the S⋯F inter­action is observed for EDOT-3 (16.1%) >> EDOT-2*A* (4.3%) > EDOT-1 (3.3%), which is also shown by the relationships of Figs. 2 ▸ and 3 ▸. For the oxygen in EDOT, O⋯H inter­actions are observed [EDOT-2*A* (8.5%) > EDOT-3 (7.2%) > EDOT-1 (2.0%)] as well as O⋯F [EDOT-2*A* (6.1%) > EDOT-3 (2.2%) > EDOT-1 (1.2%)] and O⋯Cu inter­actions [EDOT-1 (4.5%) > EDOT-2*A* and 3 (0.0%)]. These results indicate that the main inter­molecular contributions without π-inter­actions are Cu⋯O and S⋯H for EDOT-1, O⋯H for EDOT-2*A*, and S⋯F for EDOT-3.

## DFT calculations   

**Figure 5:**
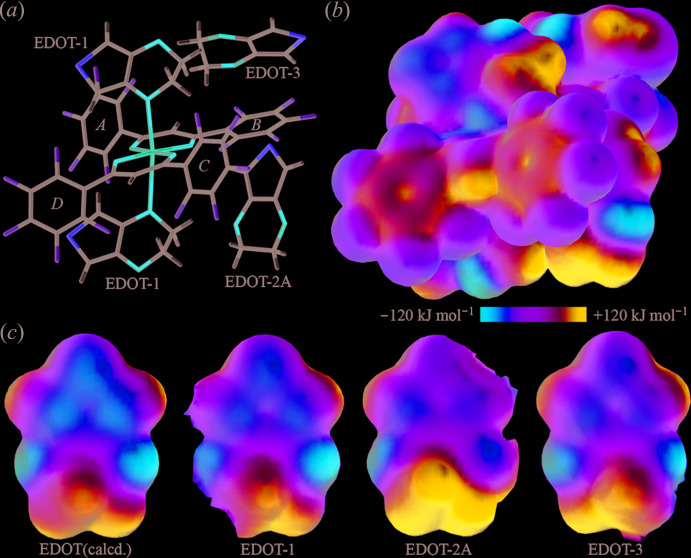
(*a*) Structure and (*b*) the energy potential maps of Cu(*L*)_2_ with the surrounding EDOT mol­ecules and (*c*) the energy potential maps of independent EDOT and each solvated EDOT mol­ecule in (I). The color of the potential is shown between −120 kJ mol^−1^ (red) to +120 kJ mol^−1^ (blue).

The DFT calculations were performed to obtain qu­anti­tative values for the surface potential and inter­molecular inter­actions. The electrostatic potentials of Cu(*L*)_2_ and EDOT in (I) range from −135.79 to +162.31 kJ mol^−1^, as shown in Fig. 5 ▸. The highest electrostatic potential, in which the electron-poor region is shown in blue, is on the Cu atom, the edge of the ketonato hydrogen, the central part of the penta­fluoro­phenyl rings in Cu(*L*)_2_, and the aromatic and aliphatic hydrogen atoms of EDOT. The lowest electrostatic potential, shown in red, is around the oxygen atoms of Cu(*L*)_2_ and EDOT. The highest electrostatic potentials of the centers of the penta­fluoro­phenyl rings *A*–*D* are approximately +97, +90, +91, +83 kJ mol^−1^, respectively, which is almost the same as the independently calculated value for Cu(*L*)_2_ (+97 kJ mol^−1^ for the penta­fluoro­phenyl ring), which was calculated using the currently reported crystal structure (Crowder *et al.*, 2019 ▸). The lowest electrostatic potentials of the five-membered rings of EDOT are −77, −63, and −63 kJ mol^−1^ for EDOT-1, 2*A* and 3, respectively, indicating the electron distribution is slightly lower than that calculated independently for EDOT (−81 kJ mol^−1^) and used to estimate the inter­molecular inter­actions of Cu(*L*)_2_ and EDOT. The electrostatic potential maps of the EDOT mol­ecules are shown in Fig. 5 ▸ *c*. The left-hand structure, optimized and calculated for an independent mol­ecule, clearly indicates that the EDOT-2*A* has more positive surfaces. The lowest electrostatic potentials of the oxygen atoms are −117 and −118 kJ mol^−1^ for EDOT (calculated from the refined structure of a single component), −85 and −121 kJ mol^−1^ for EDOT-1, −109 and −63 kJ mol^−1^ for EDOT-2*A*, and −102 and −113 kJ mol^−1^ for EDOT-3. These values show the strength of the inter­molecular inter­actions of the oxygen atoms; one oxygen in EDOT-1 is an electron donor for the coordination bond with decreasing electron density (−85 kJ mol^−1^) and one oxygen in EDOT-2*A* is an electron donor for the hydrogen bond with decreasing electron density (−63 kJ mol^−1^). The highest electrostatic potential of the surface of the aliphatic H atoms is +162 kJ mol^−1^ in EDOT-2*A* and the values of each EDOT are +116, +112, and +123 kJ mol^−1^ for EDOT (calculated), EDOT-1, and EDOT-3, respectively. The lowest electrostatic potential on sulfur is −32 kJ mol^−1^ in EDOT-2*A* and the values of each EDOT are −79, −65, and −48 kJ mol^−1^ for EDOT (calculated), EDOT-1, and EDOT-3, respectively. These results show the outflowing of the surface electrons due to the formation of the co-crystal and the corresponding inter­molecular inter­actions.

## Synthesis   

To a solution of Cu(*L*)_2_ (15 mg, 17 µmol) in chloro­form (2 ml) was added an excess amount of EDOT. The solution was evaporated slowly to give green crystals of Cu(*L*)_2_·3EDOT (I), which were separated by filtration and characterized by crystallographic and thermogravimetric (TG) analyses.

## Thermogravimetric studies   

**Figure 6:**
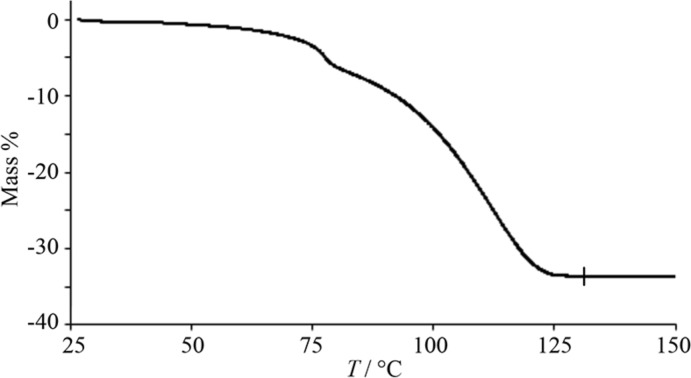
TG curves of (I) showing the one-step elimination; the scan rate was 5.0°C min^−1^.

In the TG analysis for (I), the weight loss indicates an approximate one-step elimination (Fig. 6 ▸); the total elimination of EDOT was found to be 33.6%, which is almost the same as the calculated value of 33.0% around 50–130°C. The release curve is gentle, and the coordinated EDOT and solvated EDOT are gradually separated from the crystals without being distinguished, confirming the weak coordination bond due to the Jahn–Teller effect of the Cu ion. In the complex, the positive electrostatic potential on the copper (+206.41 kJ mol^−1^) in the independent crystal of Cu(*L*)_2_ was higher than that of the corresponding non-fluorinated complex, +116.71 kJ mol^−1^ for Cu(dbm)_2_ (Kusakawa *et al.*, 2020 ▸) due to the substitution of the penta­fluoro­phenyl groups, indicating that the present EDOT recognition was induced. For the same procedure, Pd(*L*)_2_ and EDOT were combined to give brown needle-shaped crystals, which are clearly characterized as Pd(*L*)_2_ as a single component (Nakajima & Hori, 2014 ▸) and no guest release was observed by the brown crystals of Pd(*L*)_2_; the electrostatic potentials on the metal center of Pd(*L*)_2_ and Pd(dbm)_2_ are −1.0 and −73 kJ mol^−1^, respectively (Kusakawa *et al.*, 2020 ▸).

In summary, we have discussed the crystal structure and the inter­molecular inter­actions for three EDOT mol­ecules inserted in (I), in which guest recognition is induced by the flexible orientations and positive electrostatic potentials of the penta­fluoro­phenyl groups and the enhanced positive potential on the copper ion of the fluorinated complex, Cu(*L*)_2_. The crystal structure clearly suggests that the alternate coordination polymer between the metal center of Cu(*L*)_2_ and the oxygen atom of EDOT-1 was obtained along the *a* axis through the weak coordination bond and the close stacking between the penta­fluoro­phenyl group of Cu(*L*)_2_ and the aromatic moiety of EDOT-2 and EDOT-3 was obtained through the arene–perfluoro­arene inter­actions.

## Refinement   

Crystal data, data collection and structure refinement details are summarized in Table 2 ▸. H atoms were placed in geometrically idealized positions and refined as riding with C—H = 0.95 Å and *U*_iso_(H) = 1.2*U*_eq_(C) for aromatic.

## Supplementary Material

Crystal structure: contains datablock(s) global, I. DOI: 10.1107/S2056989020006155/tx2020sup1.cif

Structure factors: contains datablock(s) I. DOI: 10.1107/S2056989020006155/tx2020Isup3.hkl

CCDC reference: 2001277

Additional supporting information:  crystallographic information; 3D view; checkCIF report

## Figures and Tables

**Table 1:** Hydrogen-bond geometry (Å, °)

*D*—H⋯*A*	*D*—H	H⋯*A*	*D*⋯*A*	*D*—H⋯*A*
C23—H23⋯F17^i^	0.95	2.41	3.362 (4)	179
C31—H31⋯O1^ii^	0.95	2.57	3.351 (4)	139
C35—H35*A*⋯O8*B* ^ii^	0.99	2.45	3.349 (16)	151
C37*A*—H37*A*⋯S1^iii^	0.95	2.77	3.590 (9)	145
C41*A*—H41*A*⋯S2*A* ^iv^	0.99	2.51	3.051 (11)	114
C42*A*—H42*A*⋯S2*A* ^iv^	0.99	2.57	3.220 (9)	123
C42*A*—H42*A*⋯F6^iv^	0.99	2.45	3.162 (8)	128
C48—H48*B*⋯F10^v^	0.99	2.51	3.326 (5)	140

Symmetry codes: (i) 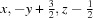; (ii) 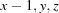; (iii) 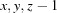; (iv) 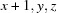; (v) 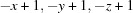.

**Table 2:** Experimental details

Crystal data	
Chemical formula	[Cu(C_15_HF_10_O_2_)_2_]·3(C_6_H_6_O_2_S)
*M*_r_	1296.36
Crystal system, space group	Monoclinic, *P*2_1_/*c*
Temperature (K)	100
*a*, *b*, *c* (Å)	7.7343 (3), 46.8973 (16), 13.2580 (5)
β (°)	99.211 (1)
*V* (Å^3^)	4746.9 (3)
*Z*	4
Radiation type	Mo *K*α
μ (mm^−1^)	0.73
Crystal size (mm)	0.17 × 0.17 × 0.11

Data collection	
Diffractometer	Bruker D8 Goniometer
Absorption correction	Multi-scan (*SADABS*; Bruker, 2018 ▸)
*T*_min_, *T*_max_	0.88, 0.93
No. of measured, independent and observed [*I* > 2σ(*I*)] reflections	54634, 8367, 7663
*R*_int_	0.042
(sin θ/λ)_max_ (Å^−1^)	0.595

Refinement	
*R*[*F* ^2^ > 2σ(*F* ^2^)], *wR*(*F* ^2^), *S*	0.049, 0.106, 1.06
No. of reflections	8367
No. of parameters	821
No. of restraints	236
H-atom treatment	H-atom parameters constrained
Δρ_max_, Δρ_min_ (e Å^−3^)	1.74, −1.69

Computer programs: *APEX3* (Bruker, 2018 ▸), *SAINT* (Bruker, 2018 ▸), *SHELXT2014/5* (Sheldrick, 2015*a* ▸), *SHELXL2018/3* (Sheldrick, 2015*b* ▸) and *shelXle* (Hübschle *et al.*, 2011 ▸).

## supplementary crystallographic information

### Crystal data

**Table:**

[Cu(C_15_HF_10_O_2_)_2_]·3(C_6_H_6_O_2_S)	*F*(000) = 2580
*M**_r_* = 1296.36	*D*_x_ = 1.814 Mg m^−^^3^
Monoclinic, *P*2_1_/*c*	Mo *K*α radiation, λ = 0.71073 Å
*a* = 7.7343 (3) Å	Cell parameters from 9808 reflections
*b* = 46.8973 (16) Å	θ = 2.6–26.4°
*c* = 13.2580 (5) Å	µ = 0.73 mm^−^^1^
β = 99.211 (1)°	*T* = 100 K
*V* = 4746.9 (3) Å^3^	Prismatic, green
*Z* = 4	0.17 × 0.17 × 0.11 mm

### Data collection

**Table:**

Bruker D8 Goniometer diffractometer	7663 reflections with *I* > 2σ(*I*)
Detector resolution: 7.3910 pixels mm^-1^	*R*_int_ = 0.042
φ and ω scans	θ_max_ = 25.0°, θ_min_ = 2.3°
Absorption correction: multi-scan (SADABS; Bruker, 2018)	*h* = −9→9
*T*_min_ = 0.88, *T*_max_ = 0.93	*k* = −55→55
54634 measured reflections	*l* = −15→15
8367 independent reflections	

### Refinement

**Table:**

Refinement on *F*^2^	236 restraints
Least-squares matrix: full	Hydrogen site location: inferred from neighbouring sites
*R*[*F*^2^ > 2σ(*F*^2^)] = 0.049	H-atom parameters constrained
*wR*(*F*^2^) = 0.106	*w* = 1/[σ^2^(*F*_o_^2^) + (0.0217*P*)^2^ + 17.5257*P*] where *P* = (*F*_o_^2^ + 2*F*_c_^2^)/3
*S* = 1.06	(Δ/σ)_max_ = 0.001
8367 reflections	Δρ_max_ = 1.74 e Å^−^^3^
821 parameters	Δρ_min_ = −1.69 e Å^−^^3^

### Special details

**Table:**

Geometry. All esds (except the esd in the dihedral angle between two l.s. planes) are estimated using the full covariance matrix. The cell esds are taken into account individually in the estimation of esds in distances, angles and torsion angles; correlations between esds in cell parameters are only used when they are defined by crystal symmetry. An approximate (isotropic) treatment of cell esds is used for estimating esds involving l.s. planes.

### Fractional atomic coordinates and isotropic or equivalent isotropic displacement parameters (Å^2^)

**Table:**

	*x*	*y*	*z*	*U*_iso_*/*U*_eq_	Occ. (<1)
C1	0.7594 (4)	0.57893 (7)	0.7738 (3)	0.0197 (7)	
C2	0.7513 (5)	0.56890 (7)	0.8708 (3)	0.0239 (7)	
C3	0.5906 (5)	0.56378 (7)	0.8994 (2)	0.0230 (7)	
C4	0.4391 (4)	0.56870 (7)	0.8317 (3)	0.0202 (7)	
C5	0.4504 (4)	0.57829 (6)	0.7348 (2)	0.0161 (6)	
C6	0.6098 (4)	0.58382 (6)	0.7030 (2)	0.0147 (6)	
C7	0.6196 (4)	0.59398 (6)	0.5964 (2)	0.0147 (6)	
C8	0.5525 (4)	0.57585 (7)	0.5164 (2)	0.0167 (6)	
H8	0.504456	0.558107	0.532651	0.020*	
C9	0.5522 (4)	0.58236 (6)	0.4136 (2)	0.0161 (6)	
C10	0.4813 (4)	0.56082 (7)	0.3334 (2)	0.0181 (7)	
C11	0.3819 (5)	0.56923 (7)	0.2409 (3)	0.0223 (7)	
C12	0.3197 (5)	0.55012 (8)	0.1642 (3)	0.0263 (8)	
C13	0.3578 (5)	0.52165 (8)	0.1781 (3)	0.0289 (8)	
C14	0.4521 (5)	0.51230 (7)	0.2688 (3)	0.0270 (8)	
C15	0.5126 (4)	0.53170 (7)	0.3443 (3)	0.0215 (7)	
C16	0.5439 (4)	0.69479 (7)	0.1554 (3)	0.0211 (7)	
C17	0.5208 (5)	0.70298 (8)	0.0544 (3)	0.0244 (7)	
C18	0.6640 (5)	0.71125 (8)	0.0115 (3)	0.0265 (8)	
C19	0.8283 (5)	0.71115 (7)	0.0703 (3)	0.0234 (7)	
C20	0.8482 (4)	0.70269 (7)	0.1712 (2)	0.0194 (7)	
C21	0.7071 (4)	0.69421 (6)	0.2165 (2)	0.0177 (7)	
C22	0.7309 (4)	0.68348 (7)	0.3249 (2)	0.0178 (7)	
C23	0.8210 (4)	0.70030 (7)	0.4022 (2)	0.0201 (7)	
H23	0.868645	0.717911	0.384186	0.024*	
C24	0.8448 (4)	0.69249 (7)	0.5051 (2)	0.0173 (7)	
C25	0.9650 (4)	0.71009 (6)	0.5807 (2)	0.0171 (7)	
C26	0.9238 (4)	0.71685 (7)	0.6765 (3)	0.0203 (7)	
C27	1.0395 (5)	0.73123 (7)	0.7492 (2)	0.0217 (7)	
C28	1.2028 (5)	0.73887 (7)	0.7288 (3)	0.0242 (8)	
C29	1.2486 (4)	0.73251 (7)	0.6353 (3)	0.0215 (7)	
C30	1.1306 (4)	0.71850 (7)	0.5624 (2)	0.0185 (7)	
Cu1	0.68190 (5)	0.63872 (2)	0.46222 (3)	0.01411 (10)	
F1	0.9178 (2)	0.58323 (5)	0.74715 (16)	0.0310 (5)	
F2	0.8979 (3)	0.56388 (5)	0.93711 (16)	0.0374 (5)	
F3	0.5815 (3)	0.55400 (5)	0.99358 (14)	0.0313 (5)	
F4	0.2828 (3)	0.56383 (5)	0.85960 (15)	0.0323 (5)	
F5	0.3000 (2)	0.58185 (4)	0.66859 (14)	0.0241 (4)	
F6	0.3354 (3)	0.59659 (4)	0.22499 (15)	0.0325 (5)	
F7	0.2237 (3)	0.55922 (5)	0.07760 (15)	0.0357 (5)	
F8	0.3024 (3)	0.50278 (5)	0.10381 (17)	0.0414 (6)	
F9	0.4876 (3)	0.48451 (4)	0.28398 (18)	0.0411 (6)	
F10	0.6066 (3)	0.52110 (4)	0.43069 (15)	0.0271 (5)	
F11	0.4006 (3)	0.68757 (5)	0.19509 (15)	0.0315 (5)	
F12	0.3608 (3)	0.70347 (5)	−0.00156 (16)	0.0382 (5)	
F13	0.6433 (3)	0.71930 (6)	−0.08624 (16)	0.0436 (6)	
F14	0.9691 (3)	0.71899 (5)	0.02976 (16)	0.0380 (6)	
F15	1.0123 (2)	0.70194 (4)	0.22413 (14)	0.0262 (4)	
F16	0.7661 (3)	0.71015 (4)	0.69951 (15)	0.0281 (5)	
F17	0.9945 (3)	0.73766 (4)	0.83999 (15)	0.0310 (5)	
F18	1.3151 (3)	0.75249 (5)	0.79938 (16)	0.0344 (5)	
F19	1.4079 (3)	0.73923 (5)	0.61553 (17)	0.0317 (5)	
F20	1.1831 (2)	0.71248 (4)	0.47335 (14)	0.0232 (4)	
O1	0.6911 (3)	0.61812 (4)	0.58985 (16)	0.0160 (5)	
O2	0.6045 (3)	0.60555 (5)	0.37963 (16)	0.0174 (5)	
O3	0.6659 (3)	0.65885 (5)	0.33475 (16)	0.0183 (5)	
O4	0.7762 (3)	0.67114 (4)	0.54204 (16)	0.0161 (5)	
C31	0.1008 (4)	0.64236 (7)	0.6576 (3)	0.0206 (7)	
H31	−0.006458	0.635978	0.676158	0.025*	
C32	0.1423 (4)	0.64075 (7)	0.5623 (2)	0.0175 (6)	
C33	0.3118 (4)	0.65181 (7)	0.5562 (2)	0.0174 (7)	
C34	0.3973 (4)	0.66157 (7)	0.6465 (3)	0.0217 (7)	
H34	0.511792	0.669515	0.656735	0.026*	
C35	0.1249 (4)	0.62454 (7)	0.3939 (3)	0.0224 (7)	
H35A	0.039407	0.621591	0.330723	0.027*	
H35B	0.194058	0.606808	0.407871	0.027*	
C36	0.2450 (4)	0.64868 (7)	0.3781 (2)	0.0203 (7)	
H36A	0.299577	0.644895	0.316742	0.024*	
H36B	0.176752	0.666565	0.366393	0.024*	
O5	0.0319 (3)	0.63026 (5)	0.47813 (17)	0.0219 (5)	
O6	0.3806 (3)	0.65200 (5)	0.46638 (17)	0.0196 (5)	
S1	0.26923 (11)	0.65754 (2)	0.74025 (6)	0.02224 (19)	
C37A	0.6149 (13)	0.63435 (13)	−0.0633 (7)	0.081 (2)	0.691 (4)
H37A	0.563149	0.637854	−0.132152	0.097*	0.691 (4)
C38A	0.8040 (15)	0.63579 (9)	−0.0290 (9)	0.065 (2)	0.691 (4)
C39A	0.8335 (12)	0.63070 (10)	0.0769 (6)	0.0505 (18)	0.691 (4)
C40A	0.6864 (13)	0.62610 (15)	0.1133 (9)	0.070 (2)	0.691 (4)
H40A	0.682749	0.622829	0.183669	0.084*	0.691 (4)
C41A	1.1061 (13)	0.6324 (2)	−0.0266 (6)	0.076 (2)	0.691 (4)
H41A	1.200067	0.640480	−0.060666	0.091*	0.691 (4)
H41B	1.116849	0.611377	−0.026951	0.091*	0.691 (4)
C42A	1.1269 (11)	0.64274 (16)	0.0798 (5)	0.0560 (18)	0.691 (4)
H42A	1.245676	0.637829	0.115366	0.067*	0.691 (4)
H42B	1.115506	0.663764	0.079751	0.067*	0.691 (4)
O7A	0.9340 (10)	0.64083 (11)	−0.0833 (4)	0.0793 (18)	0.691 (4)
O8A	1.0018 (9)	0.63079 (13)	0.1331 (6)	0.0464 (17)	0.691 (4)
S2A	0.5036 (4)	0.62657 (5)	0.0220 (3)	0.0935 (11)	0.691 (4)
C37B	0.7270 (17)	0.6447 (2)	−0.1070 (9)	0.026 (2)	0.309 (4)
H37B	0.629116	0.642764	−0.159856	0.032*	0.309 (4)
C38B	0.741 (2)	0.6349 (2)	−0.0184 (14)	0.027 (3)	0.309 (4)
C39B	0.9021 (17)	0.6392 (2)	0.0503 (10)	0.026 (2)	0.309 (4)
C40B	1.0267 (17)	0.6549 (2)	0.0030 (10)	0.033 (3)	0.309 (4)
H40B	1.142956	0.659473	0.033626	0.040*	0.309 (4)
C41B	0.644 (2)	0.6146 (4)	0.1222 (13)	0.044 (3)	0.309 (4)
H41C	0.619068	0.631998	0.159730	0.053*	0.309 (4)
H41D	0.562812	0.599481	0.138370	0.053*	0.309 (4)
C42B	0.823 (2)	0.6056 (3)	0.1583 (11)	0.048 (3)	0.309 (4)
H42C	0.854016	0.589377	0.116939	0.058*	0.309 (4)
H42D	0.835506	0.599434	0.230543	0.058*	0.309 (4)
O7B	0.6071 (13)	0.6205 (2)	0.0122 (8)	0.043 (2)	0.309 (4)
O8B	0.9395 (18)	0.6302 (3)	0.1485 (12)	0.035 (3)	0.309 (4)
S2B	0.9318 (6)	0.66369 (8)	−0.1156 (3)	0.0442 (12)	0.309 (4)
C43	0.0201 (7)	0.50447 (11)	0.7530 (4)	0.0524 (12)	
H43	−0.018735	0.523709	0.751083	0.063*	
C44	−0.0114 (5)	0.48559 (9)	0.6737 (3)	0.0328 (9)	
C45	0.0687 (5)	0.45912 (10)	0.6969 (3)	0.0356 (9)	
C46	0.1565 (7)	0.45771 (13)	0.7943 (3)	0.0626 (16)	
H46	0.218746	0.441339	0.822400	0.075*	
C47	−0.1409 (5)	0.46667 (9)	0.5206 (3)	0.0311 (9)	
H47A	−0.179232	0.472340	0.448601	0.037*	
H47B	−0.239788	0.456524	0.543732	0.037*	
C48	0.0045 (6)	0.44703 (8)	0.5246 (3)	0.0366 (9)	
H48A	−0.031048	0.430597	0.479103	0.044*	
H48B	0.103712	0.456699	0.500110	0.044*	
O9	−0.1054 (4)	0.49163 (6)	0.5800 (2)	0.0412 (7)	
O10	0.0591 (4)	0.43703 (6)	0.6276 (2)	0.0443 (7)	
S3	0.14290 (18)	0.48766 (4)	0.85819 (10)	0.0653 (4)	

### Atomic displacement parameters (Å^2^)

**Table:**

	*U*^11^	*U*^22^	*U*^33^	*U*^12^	*U*^13^	*U*^23^
C1	0.0169 (16)	0.0194 (16)	0.0221 (17)	−0.0026 (13)	0.0010 (13)	0.0019 (13)
C2	0.0243 (18)	0.0256 (18)	0.0186 (17)	−0.0002 (14)	−0.0061 (14)	0.0014 (14)
C3	0.035 (2)	0.0201 (17)	0.0139 (16)	−0.0022 (15)	0.0036 (14)	−0.0006 (13)
C4	0.0212 (17)	0.0202 (17)	0.0207 (17)	−0.0016 (13)	0.0079 (13)	−0.0019 (13)
C5	0.0164 (16)	0.0140 (15)	0.0167 (16)	0.0006 (12)	−0.0011 (12)	−0.0003 (12)
C6	0.0187 (16)	0.0098 (14)	0.0154 (15)	−0.0015 (12)	0.0020 (12)	−0.0012 (12)
C7	0.0105 (14)	0.0151 (15)	0.0182 (16)	0.0031 (12)	0.0016 (12)	0.0011 (12)
C8	0.0193 (16)	0.0120 (15)	0.0184 (16)	−0.0036 (12)	0.0022 (13)	−0.0003 (12)
C9	0.0137 (15)	0.0145 (16)	0.0197 (16)	0.0020 (12)	0.0008 (12)	−0.0023 (13)
C10	0.0191 (16)	0.0183 (16)	0.0167 (16)	−0.0003 (13)	0.0025 (13)	−0.0035 (13)
C11	0.0261 (18)	0.0197 (17)	0.0204 (17)	0.0034 (14)	0.0019 (14)	−0.0033 (14)
C12	0.0258 (19)	0.034 (2)	0.0176 (17)	0.0021 (15)	−0.0022 (14)	−0.0024 (15)
C13	0.032 (2)	0.028 (2)	0.0238 (18)	−0.0012 (16)	−0.0025 (15)	−0.0154 (15)
C14	0.032 (2)	0.0165 (17)	0.031 (2)	0.0015 (14)	0.0003 (16)	−0.0069 (15)
C15	0.0210 (17)	0.0215 (17)	0.0201 (17)	0.0011 (14)	−0.0025 (13)	−0.0037 (14)
C16	0.0208 (17)	0.0191 (17)	0.0236 (17)	−0.0019 (13)	0.0040 (14)	0.0001 (14)
C17	0.0236 (18)	0.0260 (18)	0.0217 (17)	0.0009 (14)	−0.0023 (14)	0.0026 (14)
C18	0.036 (2)	0.0291 (19)	0.0136 (16)	0.0021 (16)	0.0011 (15)	0.0077 (14)
C19	0.0261 (18)	0.0240 (18)	0.0215 (17)	−0.0026 (14)	0.0078 (14)	0.0052 (14)
C20	0.0217 (17)	0.0149 (16)	0.0204 (16)	−0.0024 (13)	0.0000 (13)	0.0005 (13)
C21	0.0250 (17)	0.0112 (15)	0.0172 (16)	−0.0025 (13)	0.0038 (13)	0.0002 (12)
C22	0.0173 (16)	0.0178 (16)	0.0188 (16)	−0.0008 (13)	0.0044 (13)	0.0012 (13)
C23	0.0242 (17)	0.0163 (16)	0.0193 (16)	−0.0065 (13)	0.0023 (13)	0.0008 (13)
C24	0.0163 (16)	0.0138 (15)	0.0226 (17)	0.0005 (12)	0.0058 (13)	−0.0029 (13)
C25	0.0213 (17)	0.0112 (15)	0.0190 (16)	−0.0029 (12)	0.0039 (13)	0.0016 (12)
C26	0.0209 (17)	0.0166 (16)	0.0243 (17)	0.0003 (13)	0.0060 (14)	−0.0007 (13)
C27	0.033 (2)	0.0153 (16)	0.0167 (16)	0.0019 (14)	0.0044 (14)	−0.0025 (13)
C28	0.0309 (19)	0.0133 (16)	0.0254 (18)	−0.0023 (14)	−0.0047 (15)	−0.0032 (14)
C29	0.0193 (17)	0.0165 (16)	0.0287 (18)	−0.0025 (13)	0.0033 (14)	0.0006 (14)
C30	0.0238 (17)	0.0137 (15)	0.0187 (16)	0.0000 (13)	0.0058 (13)	−0.0002 (13)
Cu1	0.0160 (2)	0.01277 (19)	0.01372 (19)	−0.00238 (14)	0.00286 (14)	−0.00015 (15)
F1	0.0151 (10)	0.0467 (13)	0.0298 (11)	−0.0041 (9)	−0.0011 (8)	0.0100 (10)
F2	0.0266 (11)	0.0555 (15)	0.0250 (11)	0.0015 (10)	−0.0108 (9)	0.0114 (10)
F3	0.0435 (13)	0.0369 (12)	0.0134 (9)	0.0006 (10)	0.0042 (9)	0.0057 (9)
F4	0.0260 (11)	0.0471 (13)	0.0263 (11)	−0.0014 (10)	0.0118 (9)	0.0063 (10)
F5	0.0156 (9)	0.0345 (11)	0.0211 (10)	0.0014 (8)	−0.0002 (8)	0.0049 (8)
F6	0.0462 (13)	0.0219 (11)	0.0243 (11)	0.0109 (9)	−0.0097 (9)	−0.0028 (8)
F7	0.0414 (13)	0.0422 (13)	0.0187 (10)	0.0043 (10)	−0.0100 (9)	−0.0043 (9)
F8	0.0543 (15)	0.0360 (13)	0.0294 (12)	−0.0026 (11)	−0.0068 (11)	−0.0208 (10)
F9	0.0602 (16)	0.0167 (11)	0.0412 (13)	0.0042 (10)	−0.0074 (11)	−0.0109 (10)
F10	0.0333 (11)	0.0185 (10)	0.0253 (10)	0.0031 (8)	−0.0076 (9)	−0.0014 (8)
F11	0.0209 (10)	0.0467 (13)	0.0270 (11)	−0.0044 (9)	0.0046 (9)	0.0059 (10)
F12	0.0258 (11)	0.0569 (15)	0.0278 (11)	−0.0003 (10)	−0.0077 (9)	0.0093 (11)
F13	0.0419 (14)	0.0681 (17)	0.0195 (11)	−0.0015 (12)	0.0010 (10)	0.0185 (11)
F14	0.0329 (12)	0.0576 (15)	0.0250 (11)	−0.0105 (11)	0.0098 (9)	0.0122 (10)
F15	0.0215 (10)	0.0346 (11)	0.0217 (10)	−0.0065 (9)	0.0007 (8)	0.0056 (9)
F16	0.0272 (11)	0.0320 (11)	0.0280 (11)	−0.0053 (9)	0.0137 (9)	−0.0078 (9)
F17	0.0468 (13)	0.0271 (11)	0.0201 (10)	−0.0019 (10)	0.0088 (9)	−0.0092 (9)
F18	0.0379 (13)	0.0307 (12)	0.0301 (12)	−0.0077 (10)	−0.0080 (10)	−0.0086 (9)
F19	0.0208 (11)	0.0341 (12)	0.0399 (13)	−0.0107 (9)	0.0037 (9)	−0.0027 (10)
F20	0.0252 (10)	0.0256 (10)	0.0208 (10)	−0.0040 (8)	0.0100 (8)	−0.0015 (8)
O1	0.0170 (11)	0.0142 (11)	0.0162 (11)	−0.0026 (9)	0.0014 (9)	0.0004 (9)
O2	0.0213 (12)	0.0161 (11)	0.0150 (11)	−0.0010 (9)	0.0035 (9)	−0.0015 (9)
O3	0.0220 (12)	0.0165 (11)	0.0163 (11)	−0.0056 (9)	0.0026 (9)	0.0000 (9)
O4	0.0182 (11)	0.0157 (11)	0.0154 (11)	−0.0027 (9)	0.0051 (9)	−0.0010 (9)
C31	0.0172 (16)	0.0221 (17)	0.0230 (17)	0.0004 (13)	0.0047 (13)	0.0009 (14)
C32	0.0159 (16)	0.0167 (16)	0.0194 (16)	0.0023 (13)	0.0016 (13)	−0.0002 (13)
C33	0.0151 (15)	0.0193 (16)	0.0184 (16)	0.0038 (13)	0.0051 (13)	0.0018 (13)
C34	0.0166 (16)	0.0272 (18)	0.0217 (17)	0.0008 (14)	0.0043 (13)	−0.0008 (14)
C35	0.0213 (17)	0.0259 (18)	0.0210 (17)	−0.0008 (14)	0.0063 (14)	−0.0072 (14)
C36	0.0200 (17)	0.0234 (17)	0.0172 (16)	0.0025 (13)	0.0018 (13)	−0.0010 (13)
O5	0.0159 (11)	0.0301 (13)	0.0200 (12)	−0.0033 (10)	0.0037 (9)	−0.0073 (10)
O6	0.0147 (11)	0.0276 (13)	0.0172 (11)	−0.0006 (9)	0.0046 (9)	−0.0011 (10)
S1	0.0212 (4)	0.0292 (5)	0.0160 (4)	0.0024 (3)	0.0019 (3)	−0.0007 (3)
C37A	0.100 (5)	0.031 (3)	0.083 (4)	0.006 (3)	−0.075 (4)	−0.007 (3)
C38A	0.093 (5)	0.028 (3)	0.057 (4)	−0.002 (3)	−0.042 (4)	−0.001 (3)
C39A	0.066 (4)	0.029 (3)	0.046 (4)	0.007 (3)	−0.024 (3)	−0.008 (3)
C40A	0.069 (5)	0.044 (4)	0.086 (5)	0.013 (4)	−0.022 (4)	−0.036 (4)
C41A	0.109 (5)	0.072 (4)	0.042 (4)	−0.027 (4)	−0.003 (4)	0.008 (3)
C42A	0.076 (4)	0.054 (4)	0.033 (3)	−0.014 (3)	−0.010 (3)	0.011 (3)
O7A	0.129 (4)	0.057 (3)	0.036 (3)	−0.016 (3)	−0.035 (3)	0.015 (2)
O8A	0.056 (4)	0.047 (3)	0.030 (3)	−0.003 (3)	−0.013 (3)	0.006 (2)
S2A	0.0765 (18)	0.0582 (14)	0.127 (2)	0.0261 (12)	−0.0414 (16)	−0.0615 (15)
C37B	0.033 (5)	0.028 (5)	0.017 (4)	0.013 (4)	−0.001 (4)	−0.006 (4)
C38B	0.026 (5)	0.031 (5)	0.027 (5)	0.001 (4)	0.016 (4)	−0.007 (4)
C39B	0.027 (4)	0.027 (4)	0.024 (5)	0.004 (4)	0.006 (4)	0.000 (4)
C40B	0.036 (5)	0.025 (5)	0.038 (5)	−0.001 (4)	0.002 (4)	0.001 (4)
C41B	0.050 (6)	0.054 (6)	0.033 (5)	−0.014 (5)	0.022 (5)	−0.009 (5)
C42B	0.061 (6)	0.049 (5)	0.034 (5)	−0.014 (5)	0.004 (5)	0.001 (5)
O7B	0.025 (4)	0.064 (5)	0.048 (4)	−0.018 (4)	0.033 (4)	−0.028 (4)
O8B	0.041 (6)	0.035 (5)	0.024 (5)	−0.002 (4)	−0.006 (5)	0.000 (4)
S2B	0.070 (3)	0.031 (2)	0.0374 (19)	−0.0014 (16)	0.0282 (17)	0.0000 (15)
C43	0.067 (3)	0.048 (3)	0.045 (3)	−0.010 (2)	0.017 (2)	−0.005 (2)
C44	0.031 (2)	0.036 (2)	0.030 (2)	−0.0056 (17)	0.0020 (16)	0.0027 (17)
C45	0.026 (2)	0.053 (3)	0.028 (2)	0.0078 (18)	0.0036 (16)	0.0055 (18)
C46	0.054 (3)	0.107 (5)	0.025 (2)	0.047 (3)	0.000 (2)	0.000 (2)
C47	0.0203 (18)	0.048 (2)	0.0249 (19)	−0.0103 (16)	0.0036 (15)	−0.0028 (17)
C48	0.054 (3)	0.027 (2)	0.026 (2)	−0.0040 (18)	−0.0025 (18)	0.0016 (16)
O9	0.0491 (18)	0.0391 (16)	0.0335 (15)	0.0116 (14)	0.0009 (13)	0.0044 (13)
O10	0.058 (2)	0.0410 (17)	0.0328 (16)	0.0137 (15)	0.0037 (14)	0.0049 (13)
S3	0.0512 (8)	0.1075 (12)	0.0347 (6)	0.0080 (7)	−0.0008 (5)	−0.0211 (7)

### Geometric parameters (Å, º)

**Table:**

C1—F1	1.344 (4)	C31—C32	1.355 (5)
C1—C2	1.380 (5)	C31—S1	1.717 (3)
C1—C6	1.387 (4)	C31—H31	0.9500
C2—F2	1.340 (4)	C32—O5	1.382 (4)
C2—C3	1.377 (5)	C32—C33	1.425 (4)
C3—F3	1.343 (4)	C33—C34	1.351 (5)
C3—C4	1.377 (5)	C33—O6	1.380 (4)
C4—F4	1.340 (4)	C34—S1	1.719 (3)
C4—C5	1.377 (5)	C34—H34	0.9500
C5—F5	1.350 (4)	C35—O5	1.447 (4)
C5—C6	1.390 (4)	C35—C36	1.501 (5)
C6—C7	1.505 (4)	C35—H35A	0.9900
C7—O1	1.269 (4)	C35—H35B	0.9900
C7—C8	1.393 (4)	C36—O6	1.450 (4)
C8—C9	1.396 (4)	C36—H36A	0.9900
C8—H8	0.9500	C36—H36B	0.9900
C9—O2	1.268 (4)	C37A—C38A	1.463 (14)
C9—C10	1.505 (4)	C37A—S2A	1.570 (11)
C10—C15	1.390 (5)	C37A—H37A	0.9500
C10—C11	1.396 (5)	C38A—O7A	1.347 (14)
C11—F6	1.340 (4)	C38A—C39A	1.407 (14)
C11—C12	1.383 (5)	C39A—C40A	1.323 (14)
C12—F7	1.334 (4)	C39A—O8A	1.392 (11)
C12—C13	1.373 (5)	C40A—S2A	1.708 (9)
C13—F8	1.342 (4)	C40A—H40A	0.9500
C13—C14	1.375 (5)	C41A—O7A	1.474 (11)
C14—F9	1.340 (4)	C41A—C42A	1.476 (10)
C14—C15	1.378 (5)	C41A—H41A	0.9900
C15—F10	1.349 (4)	C41A—H41B	0.9900
C16—F11	1.343 (4)	C42A—O8A	1.403 (10)
C16—C17	1.378 (5)	C42A—H42A	0.9900
C16—C21	1.387 (5)	C42A—H42B	0.9900
C17—F12	1.337 (4)	C37B—C38B	1.25 (2)
C17—C18	1.379 (5)	C37B—S2B	1.836 (14)
C18—F13	1.335 (4)	C37B—H37B	0.9500
C18—C19	1.380 (5)	C38B—O7B	1.351 (16)
C19—F14	1.341 (4)	C38B—C39B	1.44 (2)
C19—C20	1.380 (5)	C39B—O8B	1.36 (2)
C20—F15	1.348 (4)	C39B—C40B	1.433 (19)
C20—C21	1.386 (5)	C40B—S2B	1.678 (13)
C21—C22	1.505 (4)	C40B—H40B	0.9500
C22—O3	1.274 (4)	C41B—C42B	1.46 (2)
C22—C23	1.389 (5)	C41B—O7B	1.47 (2)
C23—C24	1.396 (5)	C41B—H41C	0.9900
C23—H23	0.9500	C41B—H41D	0.9900
C24—O4	1.268 (4)	C42B—O8B	1.48 (2)
C24—C25	1.501 (4)	C42B—H42C	0.9900
C25—C26	1.394 (5)	C42B—H42D	0.9900
C25—C30	1.398 (5)	C43—C44	1.365 (6)
C26—F16	1.341 (4)	C43—S3	1.745 (5)
C26—C27	1.382 (5)	C43—H43	0.9500
C27—F17	1.339 (4)	C44—O9	1.365 (5)
C27—C28	1.381 (5)	C44—C45	1.399 (6)
C28—F18	1.333 (4)	C45—C46	1.361 (6)
C28—C29	1.375 (5)	C45—O10	1.379 (5)
C29—F19	1.338 (4)	C46—S3	1.652 (6)
C29—C30	1.384 (5)	C46—H46	0.9500
C30—F20	1.339 (4)	C47—O9	1.413 (5)
Cu1—O3	1.923 (2)	C47—C48	1.448 (6)
Cu1—O4	1.928 (2)	C47—H47A	0.9900
Cu1—O1	1.940 (2)	C47—H47B	0.9900
Cu1—O2	1.941 (2)	C48—O10	1.442 (5)
Cu1—O5^i^	2.711 (2)	C48—H48A	0.9900
Cu1—O6	2.421 (2)	C48—H48B	0.9900
			
F1—C1—C2	118.4 (3)	C31—C32—O5	124.4 (3)
F1—C1—C6	119.6 (3)	C31—C32—C33	113.0 (3)
C2—C1—C6	122.0 (3)	O5—C32—C33	122.6 (3)
F2—C2—C3	119.7 (3)	C34—C33—O6	124.1 (3)
F2—C2—C1	120.8 (3)	C34—C33—C32	113.4 (3)
C3—C2—C1	119.6 (3)	O6—C33—C32	122.5 (3)
F3—C3—C4	119.9 (3)	C33—C34—S1	110.2 (3)
F3—C3—C2	120.0 (3)	C33—C34—H34	124.9
C4—C3—C2	120.2 (3)	S1—C34—H34	124.9
F4—C4—C3	120.2 (3)	O5—C35—C36	111.3 (3)
F4—C4—C5	120.5 (3)	O5—C35—H35A	109.4
C3—C4—C5	119.3 (3)	C36—C35—H35A	109.4
F5—C5—C4	118.0 (3)	O5—C35—H35B	109.4
F5—C5—C6	119.6 (3)	C36—C35—H35B	109.4
C4—C5—C6	122.4 (3)	H35A—C35—H35B	108.0
C1—C6—C5	116.6 (3)	O6—C36—C35	110.6 (3)
C1—C6—C7	121.8 (3)	O6—C36—H36A	109.5
C5—C6—C7	121.6 (3)	C35—C36—H36A	109.5
O1—C7—C8	127.3 (3)	O6—C36—H36B	109.5
O1—C7—C6	115.8 (3)	C35—C36—H36B	109.5
C8—C7—C6	117.0 (3)	H36A—C36—H36B	108.1
C7—C8—C9	123.5 (3)	C32—O5—C35	111.7 (2)
C7—C8—H8	118.2	C33—O6—C36	111.5 (2)
C9—C8—H8	118.2	C33—O6—Cu1	121.57 (18)
O2—C9—C8	125.7 (3)	C36—O6—Cu1	121.90 (18)
O2—C9—C10	115.2 (3)	C31—S1—C34	92.92 (16)
C8—C9—C10	119.1 (3)	C38A—C37A—S2A	115.0 (8)
C15—C10—C11	115.5 (3)	C38A—C37A—H37A	122.5
C15—C10—C9	123.3 (3)	S2A—C37A—H37A	122.5
C11—C10—C9	121.2 (3)	O7A—C38A—C39A	123.2 (9)
F6—C11—C12	116.9 (3)	O7A—C38A—C37A	129.5 (10)
F6—C11—C10	120.3 (3)	C39A—C38A—C37A	107.3 (11)
C12—C11—C10	122.7 (3)	C40A—C39A—O8A	126.2 (9)
F7—C12—C13	120.3 (3)	C40A—C39A—C38A	112.4 (9)
F7—C12—C11	120.4 (3)	O8A—C39A—C38A	121.4 (10)
C13—C12—C11	119.3 (3)	C39A—C40A—S2A	113.7 (9)
F8—C13—C12	120.4 (3)	C39A—C40A—H40A	123.2
F8—C13—C14	119.6 (3)	S2A—C40A—H40A	123.2
C12—C13—C14	120.0 (3)	O7A—C41A—C42A	110.5 (8)
F9—C14—C13	120.6 (3)	O7A—C41A—H41A	109.5
F9—C14—C15	119.7 (3)	C42A—C41A—H41A	109.5
C13—C14—C15	119.7 (3)	O7A—C41A—H41B	109.5
F10—C15—C14	116.6 (3)	C42A—C41A—H41B	109.5
F10—C15—C10	120.7 (3)	H41A—C41A—H41B	108.1
C14—C15—C10	122.7 (3)	O8A—C42A—C41A	111.8 (7)
F11—C16—C17	117.7 (3)	O8A—C42A—H42A	109.3
F11—C16—C21	119.8 (3)	C41A—C42A—H42A	109.3
C17—C16—C21	122.5 (3)	O8A—C42A—H42B	109.3
F12—C17—C16	120.5 (3)	C41A—C42A—H42B	109.3
F12—C17—C18	120.0 (3)	H42A—C42A—H42B	107.9
C16—C17—C18	119.5 (3)	C38A—O7A—C41A	111.8 (6)
F13—C18—C17	120.0 (3)	C39A—O8A—C42A	113.2 (7)
F13—C18—C19	120.4 (3)	C37A—S2A—C40A	91.5 (6)
C17—C18—C19	119.6 (3)	C38B—C37B—S2B	107.0 (11)
F14—C19—C20	119.7 (3)	C38B—C37B—H37B	126.5
F14—C19—C18	120.4 (3)	S2B—C37B—H37B	126.5
C20—C19—C18	119.9 (3)	C37B—C38B—O7B	120.8 (17)
F15—C20—C19	117.5 (3)	C37B—C38B—C39B	118.6 (14)
F15—C20—C21	120.5 (3)	O7B—C38B—C39B	120.6 (15)
C19—C20—C21	122.0 (3)	O8B—C39B—C40B	121.6 (13)
C20—C21—C16	116.6 (3)	O8B—C39B—C38B	126.6 (14)
C20—C21—C22	121.7 (3)	C40B—C39B—C38B	111.8 (12)
C16—C21—C22	121.6 (3)	C39B—C40B—S2B	108.2 (10)
O3—C22—C23	126.8 (3)	C39B—C40B—H40B	125.9
O3—C22—C21	114.3 (3)	S2B—C40B—H40B	125.9
C23—C22—C21	119.0 (3)	C42B—C41B—O7B	113.7 (12)
C22—C23—C24	122.9 (3)	C42B—C41B—H41C	108.8
C22—C23—H23	118.5	O7B—C41B—H41C	108.8
C24—C23—H23	118.5	C42B—C41B—H41D	108.8
O4—C24—C23	126.0 (3)	O7B—C41B—H41D	108.8
O4—C24—C25	115.4 (3)	H41C—C41B—H41D	107.7
C23—C24—C25	118.7 (3)	C41B—C42B—O8B	107.9 (13)
C26—C25—C30	116.3 (3)	C41B—C42B—H42C	110.1
C26—C25—C24	121.8 (3)	O8B—C42B—H42C	110.1
C30—C25—C24	121.7 (3)	C41B—C42B—H42D	110.1
F16—C26—C27	117.8 (3)	O8B—C42B—H42D	110.1
F16—C26—C25	120.2 (3)	H42C—C42B—H42D	108.4
C27—C26—C25	122.0 (3)	C38B—O7B—C41B	111.0 (13)
F17—C27—C28	119.7 (3)	C39B—O8B—C42B	106.6 (13)
F17—C27—C26	120.3 (3)	C40B—S2B—C37B	94.3 (6)
C28—C27—C26	120.0 (3)	C44—C43—S3	109.2 (4)
F18—C28—C29	120.2 (3)	C44—C43—H43	125.4
F18—C28—C27	120.0 (3)	S3—C43—H43	125.4
C29—C28—C27	119.7 (3)	O9—C44—C43	124.7 (4)
F19—C29—C28	120.2 (3)	O9—C44—C45	122.4 (4)
F19—C29—C30	120.0 (3)	C43—C44—C45	112.8 (4)
C28—C29—C30	119.7 (3)	C46—C45—O10	124.1 (4)
F20—C30—C29	117.2 (3)	C46—C45—C44	112.8 (4)
F20—C30—C25	120.6 (3)	O10—C45—C44	123.1 (3)
C29—C30—C25	122.2 (3)	C45—C46—S3	112.5 (4)
O3—Cu1—O4	93.47 (9)	C45—C46—H46	123.8
O3—Cu1—O1	178.39 (9)	S3—C46—H46	123.8
O4—Cu1—O1	87.48 (9)	O9—C47—C48	115.2 (3)
O3—Cu1—O2	85.82 (9)	O9—C47—H47A	108.5
O4—Cu1—O2	175.70 (9)	C48—C47—H47A	108.5
O1—Cu1—O2	93.32 (9)	O9—C47—H47B	108.5
O3—Cu1—O6	88.12 (9)	C48—C47—H47B	108.5
O4—Cu1—O6	93.75 (8)	H47A—C47—H47B	107.5
O1—Cu1—O6	90.53 (8)	O10—C48—C47	110.4 (3)
O2—Cu1—O6	90.46 (9)	O10—C48—H48A	109.6
C7—O1—Cu1	123.3 (2)	C47—C48—H48A	109.6
C9—O2—Cu1	125.0 (2)	O10—C48—H48B	109.6
C22—O3—Cu1	124.4 (2)	C47—C48—H48B	109.6
C24—O4—Cu1	123.7 (2)	H48A—C48—H48B	108.1
C32—C31—S1	110.4 (2)	C44—O9—C47	111.3 (3)
C32—C31—H31	124.8	C45—O10—C48	111.3 (3)
S1—C31—H31	124.8	C46—S3—C43	92.6 (2)
			
F1—C1—C2—F2	0.8 (5)	F16—C26—C27—F17	1.1 (5)
C6—C1—C2—F2	178.8 (3)	C25—C26—C27—F17	179.2 (3)
F1—C1—C2—C3	−178.7 (3)	F16—C26—C27—C28	−179.3 (3)
C6—C1—C2—C3	−0.7 (5)	C25—C26—C27—C28	−1.3 (5)
F2—C2—C3—F3	0.4 (5)	F17—C27—C28—F18	0.2 (5)
C1—C2—C3—F3	180.0 (3)	C26—C27—C28—F18	−179.3 (3)
F2—C2—C3—C4	−179.6 (3)	F17—C27—C28—C29	−179.4 (3)
C1—C2—C3—C4	−0.1 (5)	C26—C27—C28—C29	1.0 (5)
F3—C3—C4—F4	0.2 (5)	F18—C28—C29—F19	1.9 (5)
C2—C3—C4—F4	−179.7 (3)	C27—C28—C29—F19	−178.5 (3)
F3—C3—C4—C5	−178.9 (3)	F18—C28—C29—C30	−179.6 (3)
C2—C3—C4—C5	1.1 (5)	C27—C28—C29—C30	0.0 (5)
F4—C4—C5—F5	−1.7 (5)	F19—C29—C30—F20	−0.5 (5)
C3—C4—C5—F5	177.4 (3)	C28—C29—C30—F20	−179.0 (3)
F4—C4—C5—C6	179.5 (3)	F19—C29—C30—C25	177.6 (3)
C3—C4—C5—C6	−1.4 (5)	C28—C29—C30—C25	−0.9 (5)
F1—C1—C6—C5	178.5 (3)	C26—C25—C30—F20	178.8 (3)
C2—C1—C6—C5	0.5 (5)	C24—C25—C30—F20	4.0 (5)
F1—C1—C6—C7	−0.2 (5)	C26—C25—C30—C29	0.7 (5)
C2—C1—C6—C7	−178.2 (3)	C24—C25—C30—C29	−174.1 (3)
F5—C5—C6—C1	−178.2 (3)	C8—C7—O1—Cu1	12.6 (4)
C4—C5—C6—C1	0.6 (5)	C6—C7—O1—Cu1	−168.61 (19)
F5—C5—C6—C7	0.5 (4)	C8—C9—O2—Cu1	−4.8 (4)
C4—C5—C6—C7	179.3 (3)	C10—C9—O2—Cu1	173.90 (19)
C1—C6—C7—O1	−61.9 (4)	C23—C22—O3—Cu1	−1.0 (5)
C5—C6—C7—O1	119.4 (3)	C21—C22—O3—Cu1	177.7 (2)
C1—C6—C7—C8	117.0 (3)	C23—C24—O4—Cu1	19.4 (4)
C5—C6—C7—C8	−61.6 (4)	C25—C24—O4—Cu1	−159.1 (2)
O1—C7—C8—C9	−1.3 (5)	S1—C31—C32—O5	−178.2 (2)
C6—C7—C8—C9	179.9 (3)	S1—C31—C32—C33	0.2 (4)
C7—C8—C9—O2	−3.1 (5)	C31—C32—C33—C34	0.2 (4)
C7—C8—C9—C10	178.2 (3)	O5—C32—C33—C34	178.6 (3)
O2—C9—C10—C15	140.7 (3)	C31—C32—C33—O6	179.4 (3)
C8—C9—C10—C15	−40.5 (5)	O5—C32—C33—O6	−2.2 (5)
O2—C9—C10—C11	−37.6 (4)	O6—C33—C34—S1	−179.7 (2)
C8—C9—C10—C11	141.2 (3)	C32—C33—C34—S1	−0.5 (4)
C15—C10—C11—F6	176.1 (3)	O5—C35—C36—O6	63.7 (3)
C9—C10—C11—F6	−5.5 (5)	C31—C32—O5—C35	−165.6 (3)
C15—C10—C11—C12	−0.7 (5)	C33—C32—O5—C35	16.2 (4)
C9—C10—C11—C12	177.7 (3)	C36—C35—O5—C32	−45.5 (4)
F6—C11—C12—F7	2.4 (5)	C34—C33—O6—C36	−162.4 (3)
C10—C11—C12—F7	179.2 (3)	C32—C33—O6—C36	18.6 (4)
F6—C11—C12—C13	−177.5 (3)	C34—C33—O6—Cu1	42.2 (4)
C10—C11—C12—C13	−0.7 (6)	C32—C33—O6—Cu1	−136.9 (3)
F7—C12—C13—F8	1.6 (6)	C35—C36—O6—C33	−47.5 (3)
C11—C12—C13—F8	−178.4 (3)	C35—C36—O6—Cu1	107.8 (3)
F7—C12—C13—C14	−177.9 (3)	C32—C31—S1—C34	−0.4 (3)
C11—C12—C13—C14	2.0 (6)	C33—C34—S1—C31	0.5 (3)
F8—C13—C14—F9	−0.7 (6)	S2A—C37A—C38A—O7A	177.3 (4)
C12—C13—C14—F9	178.9 (4)	S2A—C37A—C38A—C39A	−2.5 (4)
F8—C13—C14—C15	178.5 (3)	O7A—C38A—C39A—C40A	−179.5 (3)
C12—C13—C14—C15	−1.9 (6)	C37A—C38A—C39A—C40A	0.3 (2)
F9—C14—C15—F10	0.2 (5)	O7A—C38A—C39A—O8A	0.5 (3)
C13—C14—C15—F10	−179.0 (3)	C37A—C38A—C39A—O8A	−179.7 (2)
F9—C14—C15—C10	179.7 (3)	O8A—C39A—C40A—S2A	−178.3 (4)
C13—C14—C15—C10	0.5 (6)	C38A—C39A—C40A—S2A	1.8 (4)
C11—C10—C15—F10	−179.8 (3)	O7A—C41A—C42A—O8A	62.1 (9)
C9—C10—C15—F10	1.8 (5)	C39A—C38A—O7A—C41A	16.2 (6)
C11—C10—C15—C14	0.8 (5)	C37A—C38A—O7A—C41A	−163.6 (5)
C9—C10—C15—C14	−177.6 (3)	C42A—C41A—O7A—C38A	−45.8 (8)
F11—C16—C17—F12	−0.4 (5)	C40A—C39A—O8A—C42A	−165.9 (6)
C21—C16—C17—F12	−179.4 (3)	C38A—C39A—O8A—C42A	14.1 (6)
F11—C16—C17—C18	178.4 (3)	C41A—C42A—O8A—C39A	−44.6 (8)
C21—C16—C17—C18	−0.6 (5)	C38A—C37A—S2A—C40A	3.0 (4)
F12—C17—C18—F13	−1.0 (5)	C39A—C40A—S2A—C37A	−2.8 (5)
C16—C17—C18—F13	−179.8 (3)	S2B—C37B—C38B—O7B	177.9 (5)
F12—C17—C18—C19	178.9 (3)	S2B—C37B—C38B—C39B	−2.2 (5)
C16—C17—C18—C19	0.1 (5)	C37B—C38B—C39B—O8B	179.6 (3)
F13—C18—C19—F14	−0.4 (5)	O7B—C38B—C39B—O8B	−0.5 (5)
C17—C18—C19—F14	179.7 (3)	C37B—C38B—C39B—C40B	−0.1 (3)
F13—C18—C19—C20	−179.8 (3)	O7B—C38B—C39B—C40B	179.8 (4)
C17—C18—C19—C20	0.3 (5)	O8B—C39B—C40B—S2B	−177.2 (6)
F14—C19—C20—F15	−1.9 (5)	C38B—C39B—C40B—S2B	2.5 (6)
C18—C19—C20—F15	177.5 (3)	O7B—C41B—C42B—O8B	67.4 (17)
F14—C19—C20—C21	−179.6 (3)	C37B—C38B—O7B—C41B	−171.9 (10)
C18—C19—C20—C21	−0.1 (5)	C39B—C38B—O7B—C41B	8.2 (11)
F15—C20—C21—C16	−177.9 (3)	C42B—C41B—O7B—C38B	−42.0 (16)
C19—C20—C21—C16	−0.4 (5)	C40B—C39B—O8B—C42B	−156.4 (10)
F15—C20—C21—C22	−1.0 (5)	C38B—C39B—O8B—C42B	23.9 (10)
C19—C20—C21—C22	176.6 (3)	C41B—C42B—O8B—C39B	−53.7 (14)
F11—C16—C21—C20	−178.2 (3)	C39B—C40B—S2B—C37B	−3.1 (7)
C17—C16—C21—C20	0.8 (5)	C38B—C37B—S2B—C40B	3.1 (7)
F11—C16—C21—C22	4.8 (5)	S3—C43—C44—O9	179.3 (3)
C17—C16—C21—C22	−176.2 (3)	S3—C43—C44—C45	−2.8 (5)
C20—C21—C22—O3	−124.5 (3)	O9—C44—C45—C46	179.6 (4)
C16—C21—C22—O3	52.3 (4)	C43—C44—C45—C46	1.7 (6)
C20—C21—C22—C23	54.3 (4)	O9—C44—C45—O10	−0.1 (6)
C16—C21—C22—C23	−129.0 (3)	C43—C44—C45—O10	−178.1 (4)
O3—C22—C23—C24	−3.4 (6)	O10—C45—C46—S3	−179.9 (3)
C21—C22—C23—C24	178.0 (3)	C44—C45—C46—S3	0.4 (6)
C22—C23—C24—O4	−6.9 (5)	O9—C47—C48—O10	61.2 (4)
C22—C23—C24—C25	171.6 (3)	C43—C44—O9—C47	−169.8 (4)
O4—C24—C25—C26	−42.2 (4)	C45—C44—O9—C47	12.5 (5)
C23—C24—C25—C26	139.1 (3)	C48—C47—O9—C44	−42.9 (4)
O4—C24—C25—C30	132.2 (3)	C46—C45—O10—C48	−162.8 (5)
C23—C24—C25—C30	−46.4 (4)	C44—C45—O10—C48	16.9 (5)
C30—C25—C26—F16	178.4 (3)	C47—C48—O10—C45	−44.6 (5)
C24—C25—C26—F16	−6.8 (5)	C45—C46—S3—C43	−1.7 (4)
C30—C25—C26—C27	0.4 (5)	C44—C43—S3—C46	2.6 (4)
C24—C25—C26—C27	175.1 (3)		

Symmetry code: (i) *x*+1, *y*, *z*.

### Hydrogen-bond geometry (Å, º)

**Table:**

*D*—H···*A*	*D*—H	H···*A*	*D*···*A*	*D*—H···*A*
C23—H23···F17^ii^	0.95	2.41	3.362 (4)	179
C31—H31···O1^iii^	0.95	2.57	3.351 (4)	139
C35—H35*A*···O8*B*^iii^	0.99	2.45	3.349 (16)	151
C37*A*—H37*A*···S1^iv^	0.95	2.77	3.590 (9)	145
C41*A*—H41*A*···S2*A*^i^	0.99	2.51	3.051 (11)	114
C42*A*—H42*A*···S2*A*^i^	0.99	2.57	3.220 (9)	123
C42*A*—H42*A*···F6^i^	0.99	2.45	3.162 (8)	128
C48—H48*B*···F10^v^	0.99	2.51	3.326 (5)	140

Symmetry codes: (i) *x*+1, *y*, *z*; (ii) *x*, −*y*+3/2, *z*−1/2; (iii) *x*−1, *y*, *z*; (iv) *x*, *y*, *z*−1; (v) −*x*+1, −*y*+1, −*z*+1.
